# *Lactobacillus acidophilus* alleviates the inflammatory response to enterotoxigenic *Escherichia coli* K88 via inhibition of the NF-κB and p38 mitogen-activated protein kinase signaling pathways in piglets

**DOI:** 10.1186/s12866-016-0862-9

**Published:** 2016-11-10

**Authors:** Haihua Li, Lei Zhang, Longbin Chen, Qi Zhu, Wenjie Wang, Jiayun Qiao

**Affiliations:** Tianjin Institute of Animal Husbandry and Veterinary Medicine, Tianjin, China

**Keywords:** ETEC, Inflammatory response, Innate immunity, *L. acidophilus*, Mitogen-activated protein kinase, Nuclear factor kappa B, Toll-like receptor

## Abstract

**Background:**

A newly isolated *L. acidophilus* strain has been reported to have potential anti-inflammatory activities against lipopolysaccharide (LPS) challenge in piglet, while the details of the related inflammatory responses are limited. Here we aimed to analysis the ability of *L. acidophilus* to regulate inflammatory responses and to elucidate the mechanisms involved in its anti-inflammatory activity.

**Results:**

The ETEC (enterotoxigenic *Escherichia coli*) K88-induced up-regulations of IL-1β, IL-8 and TNF-α were obviously inhibited by *L. acidophilus* while IL-10 was significantly increased*.* Moreover, *L. acidophilus* down-regulated pattern recognition receptors TLR (Toll-like receptor) 2 and TLR4 expression in both spleen and mesenteric lymph nodes of ETEC-challenged piglets, in accompanied with the reduced phosphorylation levels of nuclear factor kappa B (NF-κB) p65 and mitogen-activated protein kinase (MAPK) p38 as well in spleen of ETEC-infected piglets. Furthermore, *L.acidophilus* significantly increased the expression of the negative regulators of TLRs signaling, including Tollip, IRAK-M, A20 and Bcl-3 in spleen of ETEC-challenged piglets.

**Conclusions:**

Our findings suggested that *L. acidophilus* regulated inflammatory response to ETEC via impairing both NF-κB and MAPK signaling pathways in piglets.

## Background

Probiotics contribute to maintaining a healthy digestive system of the host via regulating both intestinal development and flora balance [1–3], some of which have been shown to enhance the host immune responses by regulating cytokine and chemokine production [2]. *L. acidophilus* strain is a well-characterized probiotic bacterium, which has been reported to improve the production performance of animals as well as enhance the immune responses [4–6]. TLRs are essential for triggering the innate immune response by sensing pathogen-associated molecular patterns (PAMPs) [2, 7], which activates nuclear factor kappa B (NF-κB) and mitogen-activated proteinkinase (MAPK) signaling pathway [8, 9].

In our previous study, a newly isolated *L. acidophilus* strain has shown potential protective activity against inflammatory response to lipopolysaccharide (LPS) in piglets [4], but the underlying molecular mechanism is still unknown. LPS present in the outer membranes of some Gram-negative pathogens, such as ETEC, which can trigger the production of proinflammatory mediators that may contribute to intestinal inflammation and consequent inflammatory damages during the infection [9–11]. *Escherichia coli* (*E. coli*) is one of the predominant species of facultative anaerobes in the pig gut and an opportunistic pathogen to the host. ETEC, as a group of pathogenic *E. coli*, can causes diarrheal disease in pigs [10, 12]. Therefore, here we use piglets infected with ETEC as experimental models to investigate the role of *L. acidophilus* in both NF-κB and MAPK signaling pathways, thus to determine the regulative ability of *L. acidophilus* on inflammatory responses and deepen the mechanisms involved in reduced inflammation during ETEC infection by *L. acidophilus.*

## Methods

### Bacterial strain

ETEC strain K88 from the China Veterinary Culture Collection Center was grown in Luria-Bertani (LB) broth containing 1 % tryptone and 0.5 % yeast extract (both from OXOID) plus 1 % NaCl, pH 7.0. After incubation at 37 °C with vigorous shaking overnight, bacteria were 1:100 diluted in fresh LB and grown for 2 h. The bacterial cells were harvested by centrifugation at 3000 × g for 10 min at 4 °C, washed in 0.9 % NaCl solution and resuspended in saline.

The *L. acidophilus* was grown in MRS (De Man, Rogosaand Sharpe) medium at 37 °C under anaerobic environment. Culture solution of the strain was centrifuged at 3000 × g for 10 min at 4 °C. Bacterial powder was acquired according to the treatment in a vacuum freeze-drying machine (Tofflon, Shanghai, China), and there are 5 × 10^10^ CFU/g *Lactobacillus* in freeze-drying powder. Bacterial concentrations of both ETEC and *Lactobacilli* were determined in preliminary experiments by densitometry and confirmed by serial dilutions followed by CFU counts of ETEC on LB agar after 16-h incubation and the lactobacilli on MRS agar after 48-h incubation under anaerobic environment.

### Animals and experimental design

All pigs, which were purchased from Tianjin Nongfu Agriculture and Animal Husbandry Co. Ltd, used in this experiment were born naturally at full term (114 days of gestation). A total of 12 crossbred healthy female piglets (Duroc × Landrace × Yorkshiere) were reared by sows and weaned at 21 ± 2 days of age. After a 7-day period of adaptation, the pigs (5.34 ± 0.09 kg) were allotted to 1 of 4 dietary treatments (3 pigs per treatment). *L. acidophilus* were included in the diet by replacing the same amount of corn. The corn-soybean meal-fish meal basal diet (Table 1) was formulated to meet the National Research Council (NRC 2012) requirements for all nutrients.

**Table 1:** Ingredient and chemical composition of basal diets (% w/w, as-fed basis)

Item	Amount
Corn, yellow	63.20
Soybean meal, 43 % CP (crude protein)	19.00
Whey powder	4.80
Fish meal, 65 % CP	8.60
Glucose	1.00
Acidifier	0.30
Calcium hydrogen phosphate	0.60
Limestone	0.70
Salt	0.30
L-Lys•HCL, 78 % Lys	0.30
DL-Met, 99 % Met	0.10
L-Thr, 98 % Thr	0.10
Vitamin and mineral premix^a^	1.00
Calculated composition	
DE (digestible energy), Mcal/kg	3.25
Lys, %	1.39
Met, %	0.53
Analyzed composition,	
Crude protein	18.75
Crude fat	3.42
Calcium	0.88
Total phosphorus	0.71
Crude fiber	2.20

^a^Supplying a minimum per kilogram complete diet of: 12,500 IU Vitamin A; 1250 IU Vitamin D; 125 IU Vitamin E; 90 μg Vitamin B_12_; 10 mg riboflavin; 48 mg pantothenic acid; 35 mg niacin; 4.5 mg folic acid; 0.25 mg biotin; 130 mg Fe; 180 mg Zn; 15 mg Cu; 30 mg Mn; 0.60 mg I and 0.25 mg Se

The experiment was arranged as a 2 × 2 factorial arrangement of 2 diets (basal diet with or without *L. acidophilus*) and subsequently ETEC challenged (pigs challenged with ETEC or treated with sterile saline). (1) Control group (piglet fed the basal diet and receiving oral administration of 0.9 % NaCl solution); (2) ETEC group (piglet fed the basal diet and receiving oral administration of ETEC); (3) *L. acidophilus* group (piglets fed the basal diet supplemented with 0.2 % *L. acidophilus* powder and receiving oral administration of 0.9 % NaCl solution); (4) ETEC + *L. acidophilus* group (piglets fed the basal diet supplemented with 0.2 % *L. acidophilus* powder and receiving oral administration of ETEC).

Each pen was equipped with a feeder and a nipple water to allow piglets free access to feed and drinking water, and maintained at ambient temperature of 20 ~ 30 °C. All piglets had free access to the basal diet (Table 1) between 21 and 28 days of age for adapting to solid food, and pigs were received the four diets respectively at 28 days of age throughout the 14-d feeding trial. At 42 days of age, the challenged group was orally received *E. coli* K88 at 1 × 10^9^ CFU/kg BW and the unchallenged group was orally received with the same amount of 0.9 % NaCl solution. The dosage of ETEC was chosen according to Li et al. [13]. ETEC (1 × 10^9^ CFU/mL) was diluted in sterile 0.9 % NaCl.

### Blood and tissue sample collections

Three hours after the ETEC or saline treatment, blood samples (5 ml per piglet) of piglets were collected through precava. Serums were obtained by centrifugation at 3000 rpm and 4 °C for 20 min and stored at −20 °C before analysis. The spleen and MLNs samples were harvested by scraping with a glass slide, immediately frozen in liquid nitrogen, and then stored at −80 °C for further analysis. According to the previous reports, ETEC can induce acute inflammatory responses within 1–6 h following the oral challenge in piglets, which results in intestinal morphologic damage and the impairment of intestinal barrier function [14–16]. Therefore, the time point of 3 h following ETEC or saline treatment was chosen for experimental measurements.

### Detection of serum cytokine levels by ELISA

The concentrations of IL-1β, IL-8, TNF-α and IL-10 in serums were measured using commercially available ELISA kits specific for porcine IL-1β, IL-8, TNF-α and IL-10 (R and D Systems, Minneapolis, MN) according to the manufacturer’s instructions.

### RNA extraction and real-time RT-PCR

Total RNA was extracted from tissue samples using the TRIzol reagent [TaKaRa Biotechnology (Dalian)] according to the manufacturer’s guidelines, and 1 μg of RNA was used for cDNA synthesis using Moloney murine leukemia virus (MMLV; Promega) to examine expression of TLR2, TLR4, Tollip, IRAK-M, A20 and Bcl-3. Real-time PCR was performed using specific primers for TLR2 [7] (sense 5′-TCA TCT CCC AAA TCT GCG AAT-3′, antisense 5′-GGC TGA TGT TCT GAA TTG ACCTC-3′), TLR4 [7] (sense 5′-CCG TCA TTA GTG CGT CAG TTCT-3′, antisense 5′-TTG CAG CCC ACA AAA AGCA-3′), Tollip [9] (sense 5′-TAC CGT GGG CCG TCTCA-3′, antisense 5′-CCG TAG TTC TTC GCC AAC TTG-3′), IRAK-M [9] (sense 5′-TGG AGC AGC CTT GAA TCCTT-3′, antisense 5′-TGG ATA ACA CGT TTG GGA ATCTT-3′), A20 [9] (sense 5′-CCT CCC TGG AAA GCC AGAA-3′, antisense 5′-GTG CCA CAA GCT TCC TCA CTT-3′), Bcl-3 [9] (sense 5′-CGA CGC GGT GGA CAT TAAG-3′, antisense 5′-ACC ATG CTA AGG CTG TTG TTT TC-3′), or β-actin [9] (sense 5′-CAT CAC CAT CGG CAA CGA-3′, antisense 5′-GCG TAG AGG TCC TTC CTG ATGT-3′) and the Real Time SYBR master mix kit [TaKaRa Biotechnology (Dalian)] in accordance with the manufacturer’s protocol for the ABI7500 real-time PCR system (Applied Biosystems, Life Technologies). Gene expression was normalized to β-actin (internal reference) and presented as relative fold change compared with control group. All samples were run in triplicate.

### Detection of both NF-κB and p38 MAPK activation by western blot

The tissue samples (50–100 mg) were homogenized in 1 mL of NP-40 lysis buffer supplemented with protease and phosphatase inhibitors and centrifuged at 12,000 × g for 15 min at 4 °C. Tissue proteins were separated by 12 % sodium dodecyl sulfate-polyacrylamide gel electrophoresis (SDS-PAGE) and transferred onto the nitrocellulose membranes. After blocking with 3 % BSA in Tris-buffered saline including 0.1 % Tween-20 buffer, membranes were incubated with primary antibodies against NF-κBp65 (Cell Signaling), phospho-NF-κBp65 (Cell Signaling), p38 MAPK (Cell Signaling), phospho-p38 MAPK (Cell Signaling) or β-actin (Sigma-Aldrich). Membranes were then washed and incubated with appropriate secondary antibodies. Proteins were visualized using the ECL reagent according to the manufacturer’s instructions. One sample from each treatment was run in duplicate in a gel and 12 samples from 4 treatments (*n* = 3) were run in 3 gels at one time to minimize the variations of gel to gel. Densitometric analysis was performed by Gel-Pro analyzer and normalized to β-actin.

### Statistical analysis

Data were evaluated using one-way ANOVA (SAS Institute Inc., Cary, NC, 2002). The Tukey multiple comparison test was used to determine differences among the means of treated groups. A probability value of <0.05 was considered statistically significant.

## Results

### *L. acidophilus* regulates ETEC-induced serum cytokine production in piglet

**Fig. 1:**
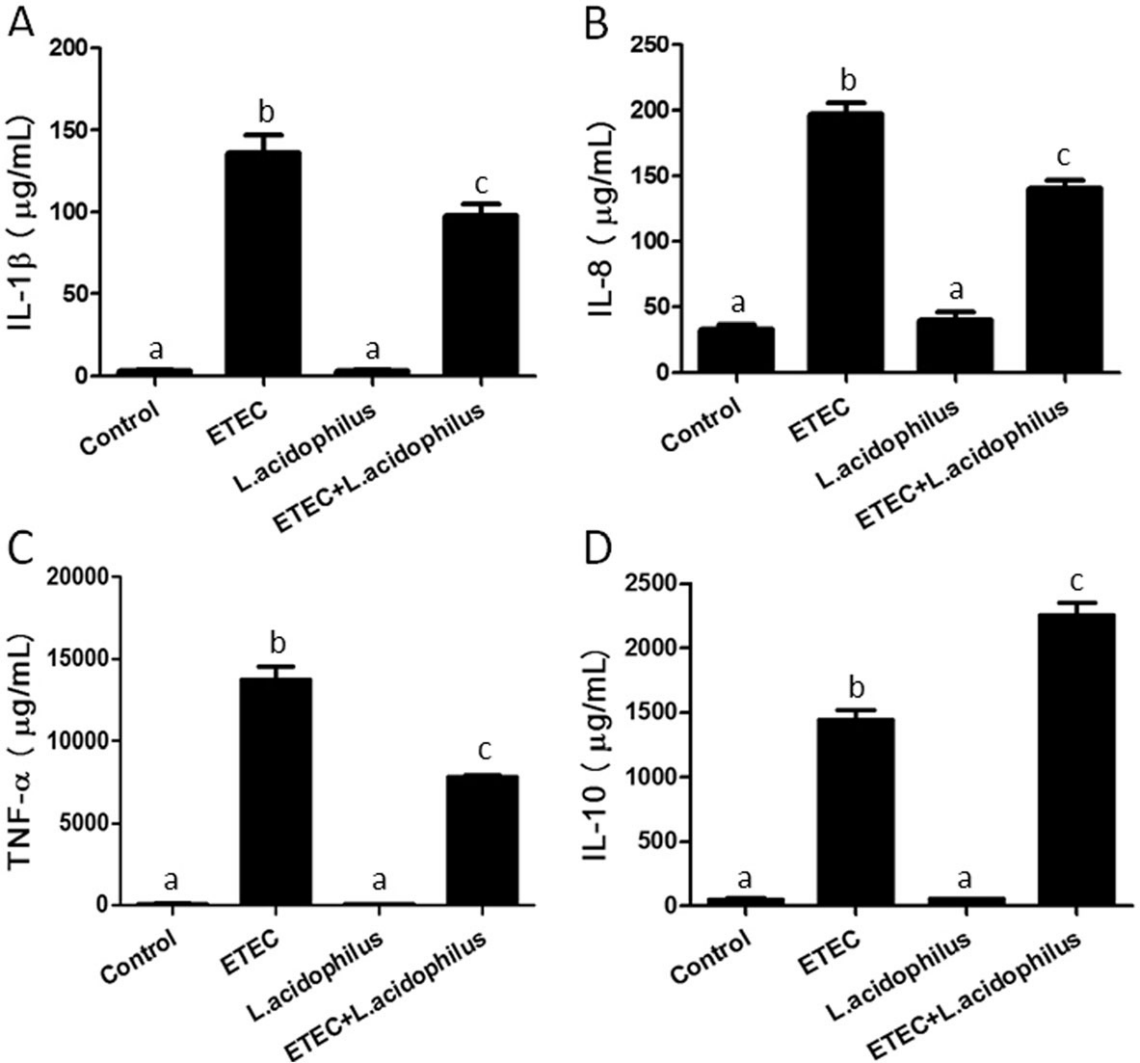
*L. acidophilus* regulates ETEC-induced serum cytokine production in piglet. Pigs were challenged with or without ETEC (1 × 10^9^ CFU/kg BW), the levels of IL-1β (**A**), IL-8 (**B**), TNF-α (**C**) and IL-10 (**D**) proteins in serums were evaluated 3 h after the inflammatory challenge. Values with different letters are significantly different (*P* < 0.05). (*n* = 3)

In our previous study, we evaluated the anti-inflammatory effect of *L. acidophilus* in piglets challenged with LPS, and established the appropriate dose of *L. acidophilus* (0.2 %) for further studies on inflammatory responses [4]. Here we used the same dose of oral administration to evaluate anti-inflammatory function of *L. acidophilus* in piglets challenged with ETEC. We found that serum levels of IL-1β (Fig. 1a), IL-8 (Fig. 1b), TNF-α (Fig. 1c) and IL-10 (Fig. 1d) increased significantly in ETEC group compared with Control group. On the contrary, the proinflammatory cytokines which were increased in ETEC group, such as IL-1β (Fig. 1a), IL-8 (Fig. 1b) and TNF-α (Fig. 1c), were suppressed in ETEC + *L. acidophilus* group, while the significant increase of anti-inflammatory cytokine IL-10 was observed (Fig. 1d). Meanwhile, the uptake of 0.2 % *L. acidophilus* (*L. acidophilus* group) had no effects on the production of the indicated cytokines (Fig. 1a-d). These results indicated that we established an appropriate model to study the anti-inflammatory mechanism of *L. acidophilus.*

### *L. acidophilus* down regulates ETEC-induced Toll-like receptors mRNA expression in spleen and MLNs

**Fig. 2:**
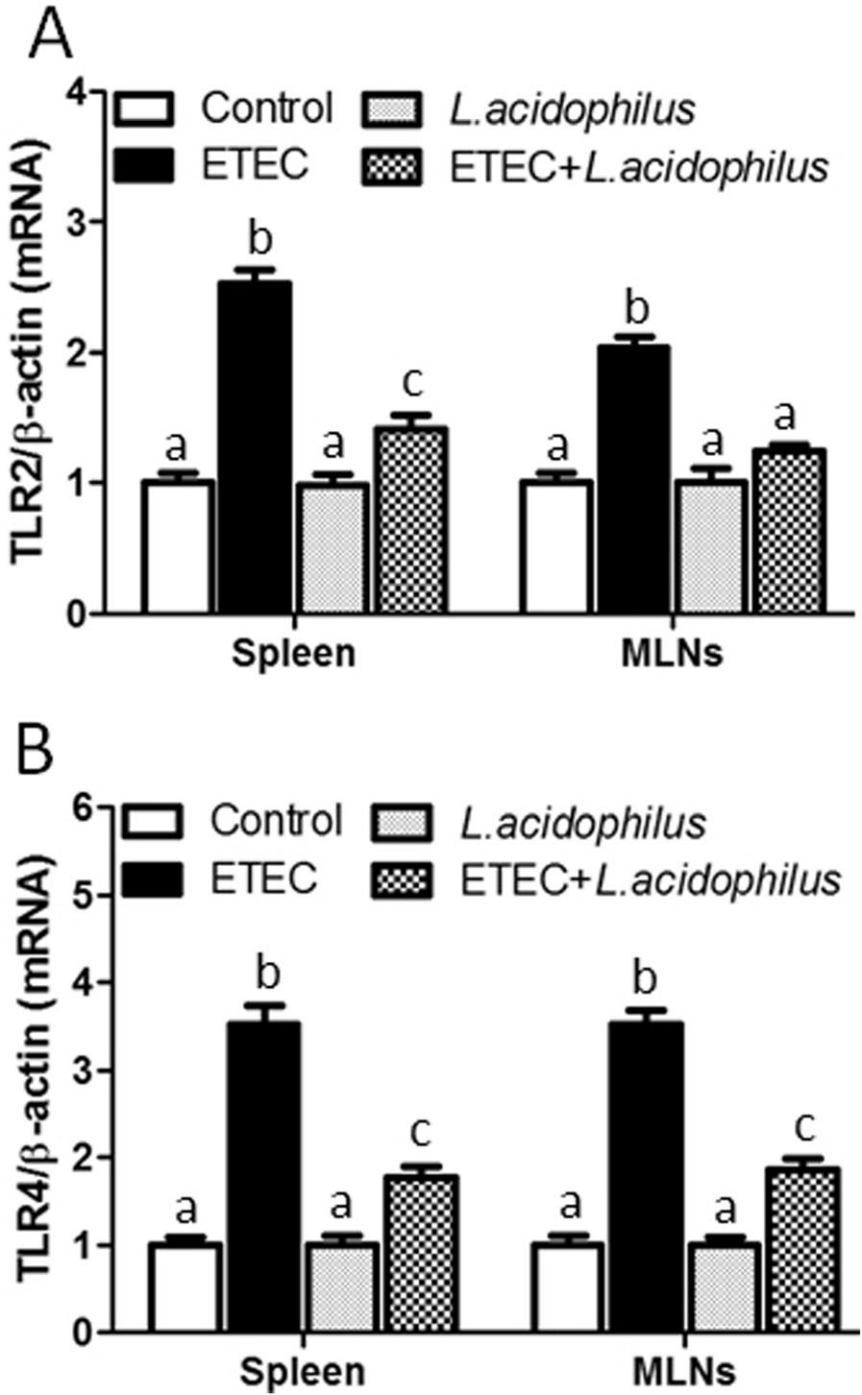
*L. acidophilus* impaired ETEC-trigged TLR2 and TLR4 expression in spleen and MLNs. Pigs were challenged with or without ETEC (1 × 10^9^ CFU/kg BW). Three hours post inflammatory challenge, spleen and MLNs samples were collected to examine the levels of porcine TLR2 (**A**) and TLR4 (**B**) by real-time RT-PCR. Expression of TLR2 and TLR4 was normalized to β-actin in the same sample, and presented as fold change relative to mock challenged pigs fed with basal diet (Control group). Expression of TLR2 or TLR4 in control group was normalized to 1.0. Values with different letters are significantly different (*P* < 0.05). (*n* = 3)

TLRs are key components that trigger the innate immune response against pathogens. Mature lymphocytes and macrophages expressing TLRs were settled in the surrounding lymphoid organs such as spleen and mesenteric lymph nodes (MLNs). TLR2 and TLR4 play important roles in the inflammatory responses to different pathogenic bacteria [12, 17]. This prompted us to investigate whether the TLR2 and TLR4 are involved in inflammatory regulatory function of *L. acidophilus* in ETEC-challenged piglets. We examined the mRNA levels of both TLR2 and TLR4 in spleen and MLNs using real-time RT-PCR. There was no difference between *L. acidophilus* group and the control. As shown in Fig. 2a, the levels of TLR2 increased 1.5-fold and 1.0-fold in spleen and MLNs respectively, and the levels of TLR4 increased 2.5-fold both in spleen and MLNs (Fig. 2b), in ETEC group compared with control group. However, we found down-regulation 44 % and 39 % of the level of TLR2 in spleen and MLNs respectively (Fig. 2a), and 50 and 47 % of the level of TLR4 in spleen and MLNs respectively (Fig. 2b) in ETEC + *L. acidophilus* group compared with ETEC group. The data indicated that *L. acidophilus* down-regulated the ETEC-induced Toll-like receptors expression in mRNA levels.

### *L. acidophilus* inhibited NF-κB and p38 MAPK activation in ETEC-challenged piglet in spleen

**Fig. 3:**
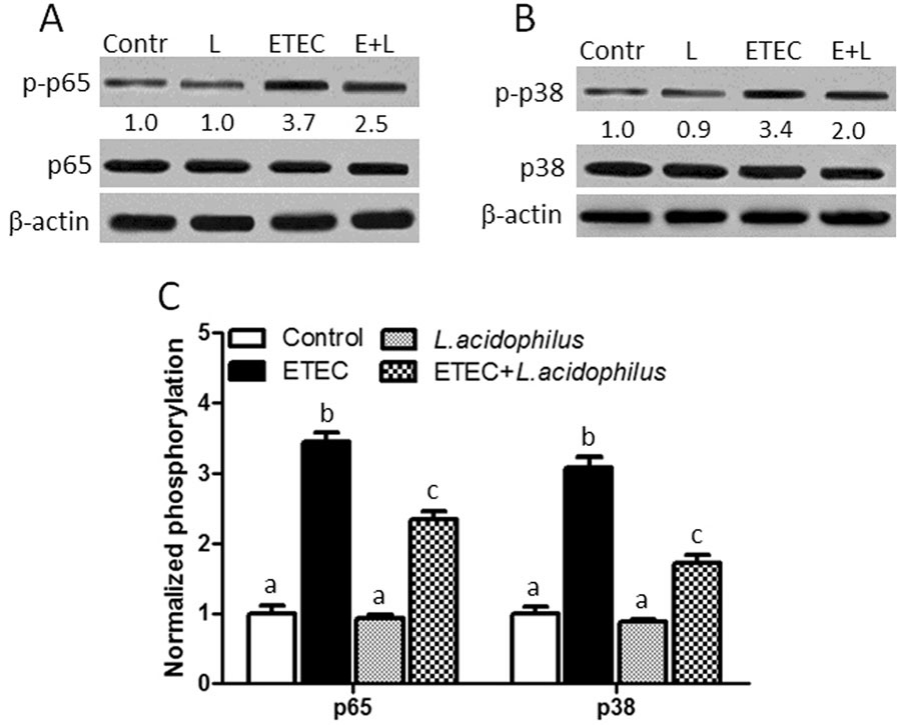
*L. acidophilus* inhibited NF-κB and p38 MAPK activation in spleen of ETEC-challenged piglet. Pigs were challenged with or without ETEC (1 × 10^9^ CFU/kg BW). Three hours post inflammatory challenge, spleen samples were collected to examine the expression of p65 (**A**), p-p65 (**A**), p38 (**B**) and p-p38 (**B**) by western blotting. Densitometric analysis was performed and normalized to β-actin (**C**). Phosphorylation of p65 or p38 in control group was normalized to 1.0. Values with different letters are significantly different (*P* < 0.05). (*n* = 3)

Activation of TLRs activates NF-κB and MAPK signaling, which are the immune-related transcriptional factors that stimulate synthesis of cytokines and chemokines [2, 8, 9]. It has been reported that ETEC or LPS is able to induce NF-κB and MAPK activation in porcine intestinal epithelial cell line (PIE cells) [9]. We therefore evaluated the effect of *L. acidophilus* on NF-κB and MAPK activation during ETEC oral challenge. Levels of p65, p-p65, p38, and p-p38 proteins in spleen were measured by western blotting (Fig. 3a and b). As shown in Fig. 3c, the phosphorylation levels of p65 and p38 in ETEC group were significantly increased compared with control group. *L. acidophilus* group did not modify the level of p-p65 and p-p38 compared with control group. However, ETEC + *L. acidophilus* group abrogated the increase of p-p65 and p-p38 level compared with ETEC group. These results indicated that *L. acidophilus* can counteract the ETEC-induced NF-κB and MAPK activation.

### *L. acidophilus* upregulated negative regulators of TLR signaling pathway in ETEC-infected piglet in spleen

**Fig. 4:**
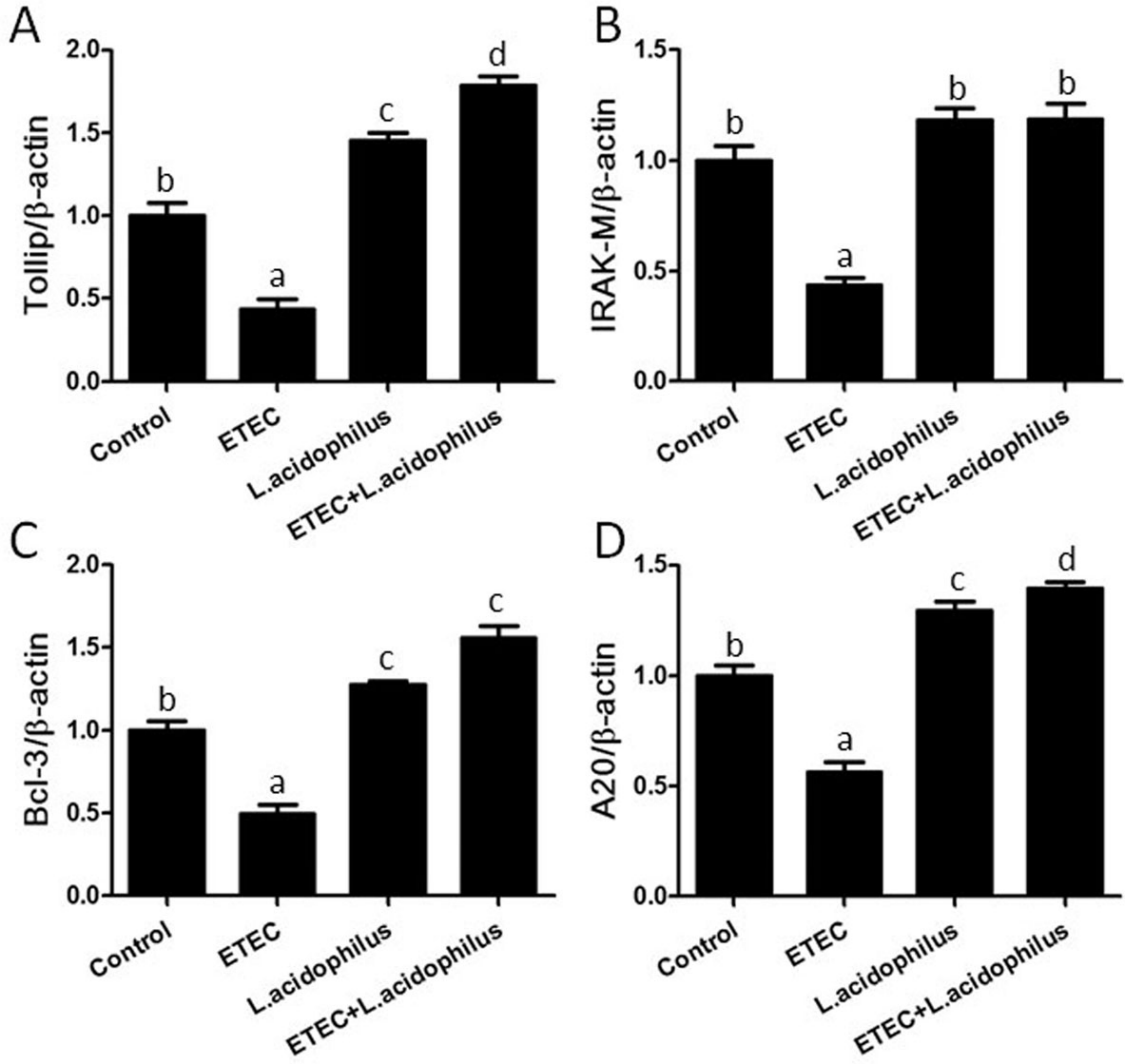
*L. acidophilus* upregulated negative regulators of TLRs signaling pathway in spleen of ETEC-infected piglet. Pigs were challenged with or without ETEC (1 × 10^9^ CFU/kg BW). Three hours post inflammatory challenge, spleen samples were collected to examine the levels of Tollip (**A**), IRAK-M (**B**), Bcl-3 (**C**) and A20 (**D**) by real-time RT-PCR. Expression of Tollip, IRAK, A20 and Bcl-3 was normalized to β-actin in the same sample, and presented as fold change relative to ETEC-challenged pigs fed with basal diet (ETEC group). Expression of Tollip, IRAK, A20 or Bcl-3 in ETEC group was normalized to 1.0. Values with different letters are significantly different (*P* < 0.05). (*n* = 3)

We also studied the role of negative regulators which mediates the TLR signaling pathway on relieved inflammation by *L. acidophilus* in ETEC infection. The expression levels of Toll interacting protein (Tollip), interleukin-1 receptor-associated kinase M (IRAK-M), A20 and B-cell lymphoma 3-encodedprotein (Bcl-3) in spleen were determined using real-time RT-PCR. We found that ETEC group exhibited obviously lower expression of Tollip (Fig. 4a), IRAK-M (Fig. 4b), A20 (Fig. 4c) and Bcl3 (Fig. 4d) compared with the control group, but higher levels of Tollip (Fig. 4a), A20 (Fig. 4c) and Bcl-3 (Fig. 4d) were shown in *L. acidophilus* group, compared with the control group. We also found that “ETEC + *L. acidophilus*” group showed obviously higher expression of Tollip (Fig. 4a), IRAK-M (Fig. 4b), A20 (Fig. 4c) and Bcl3 (Fig. 4d) compared with *L. acidophilus* group. As our expected, there was extreme difference between “ETEC + *L. acidophilus*” group and ETEC group. The present data indicated that infection with ETEC strongly downregulated the levels of mRNA expression of the TLR antagonists, and this effect was impaired by *L. acidophilus*.

## Discussion

It is demonstrated that Lactic acid bacteria (LAB) are beneficial in the treatment of a variety of mucosal disorders including inflammatory damages even in the absence of infection [9, 18–20], especially during the period of weaning [12]. Studies that evaluated the effects of probiotic strains on immune responses demonstrate that probiotic-mediated protection against pathogen-induced inflammation, in part, from maintaining the balance of proinflammatory and anti-inflammatory cytokines production in immune cells [12, 21–24]. *L. acidophilus*, one of the popular probiotics, has been confirmed its role of inflammatory regulation in human MKN45 cells [22], intestinal epithelial cells [23] and in piglets [4]. Based on the increasingly supporting evidence of the beneficial effects of probiotics, it is important to explore the molecular mechanism of LAB-regulated inflammatory reaction. In this study, we used piglets orally challenged by ETEC K88 as experimental models to investigate the function of *L. acidophilus* in inflammatory response thus to elucidate the mechanisms involved in anti-inflammatory activities.

Many cytokines are of importance to influence the intestinal function [25, 26]. For examples, IL-8 has been associated with pathogen-induced alterations of tight junctions [25, 26], as well as TNF-α has remarkable functional duality that is strongly engaged in both tissue regeneration and destruction [13]. Several studies in vitro have shown that *L. acidophilus* is able to modulate the production of proinflammatory cytokines, including IL-8, TNF-α and IL-6 in intestinal epithelial cells [8, 12, 21, 23, 27]. Here, we observed significant down-regulated proinflammatory cytokines by *L. acidophilus* including IL-1β, IL-8 and TNF-α in piglets challenged with ETEC and a remarkable rise of anti-inflammatory cytokine IL-10 production*.* This result was consistent with our previous findings that *L. acidophilus* alleviated inflammatory response to LPS in piglets [4]. Referring to the weakening effects on pathogenic bacteria-induced inflammation of lactic acid bacteria, some studies in vitro have shown that *L. plantarum* and *L. rhamnosus* suppress *E.coli*-induced proinflammatory cytokines of IL-1α, IL-8 and TNF-α expression [13, 28], and *L. acidophilus* can decrease Salmonella-induced TNF-α and IL-8 in human intestinal Caco-2 cells [29]. Our findings indicate that the *L. acidophilus* can also counteract the inflammation induced by a pathogenic bacterium, ETEC, in weaned piglets.

TLR2 and TLR4 are well known to initiate the inflammatory response to pathogenic bacteria [12, 17]. Intestinal damage caused by inflammation can be mediated by the inflammatory response triggered by the interaction between pathogens and TLRs. Inappropriate TLR signaling can lead to impaired tolerance to PAMPs which results in sever intestinal epithelium injury [9, 30]. TLR2 and TLR4 are believed to favor signaling the production of inflammatory cytokines [24]. Thus, it prompted us to investigate whether TLR2 and TLR4 are involved in inflammatory response to ETEC and can be regulated by *L. acidophilus*. Our data indicated that ETEC infection stimulated the expression of TLR2 and TLR4 in both spleen and MLNs in ETEC-challenged piglets, which were significantly down-regulated by the presence of *L. acidophilus* in the diet. Similar results were found in other LAB. *L. plantarum* N14 (LP14) regulates the production of proinflammatory cytokines from PIEs in response to ETEC challenge via TLR2 and TLR4 [21]. *L. delbrueckii* subsp. delbrueckii TUA4408L (Ld) suppress inflammatory cytokines production through TLR2 in PIE cells, and its acidic extracellular polysaccharide (APS) plays the role of immunomodulatory action by TLR4 [28]. *﻿L. jensenii* TL2937 weakened the levels of proinflammatory cytokines and chemokines triggered by ETEC or LPS challenge by decreasing TLR4 expression [9]. Our findings provide evidence that both TLR2 and TLR4 signaling play important role of the inflammatory regulation of *L. acidophilus* during ETEC infection.

TLRs signal the activation of NF-κB and MAPK via multiple downstream intracellular factors [2, 24]. Activated NF-κB and MAPK stimulate synthesis of anti- and proinflammatory cytokines, including IL-10, IL-1β, TNF-a, IL-6 and IL-8, etc. [3, 8, 22–24, 31]. Thus, it will be interesting to explore whether NF-κB and MAPK are involved in *L. acidophilus*-mediated regulation on inflammatory response to ETEC*.* As our expected, *L. acidophilus* decreased the phosphorylation levels of both NF-κB and p38 MAPK in spleen from ETEC-infected piglets. Probiotic *L. acidophilus* is reported to decrease Salmonella-induced NF-κB activation in human intestinal Caco-2 cells [30]. Experiments in demonstrated that *L. plantarum* N14 (LP14) strongly activated NF-κB via RP105 and TLR2 in ETEC-infected PIE cells [21]. Ld strain and its APS can inhibit ETEC triggered mitogen-activated protein kinase (MAPK) and nuclear factor-κB (NF-κB) activation in PIE cells [28]. In addition, *L. jensenii* TL2937 weakened the levels of proinflammatory cytokines and chemokines induced by ETEC or LPS treatment via down-regulating NF-κB and MAPK activation [9]. Therefore, it is likely that the *L. acidophilus* alleviates the ETEC-induced inflammatory response through weakening the activation of these transcriptional factors of host cells.

The immune system needs to constantly communicate to maintain homeostasis between activation and inhibition of TLRs signals to avoid inflammatory immunological imbalance [32]. Negative regulatory mechanisms are important to weaken TLR signaling and keep the intact signaling pathway. In this regard, a ever increasing number of inhibitory factors of TLR signaling, including Tollip, IRAK-M, A20, Bcl-3 and peroxisome proliferator-activatedreceptor-γ (PPARγ) are found and characterized, which guard against chronic inflammatory and potentially detrimental TLR responses to microbe-associated molecular patterns (MAMPs) from intestinal commensal bacteria [33]. Acturally, commensal gut bacteria can regulate TLR negative regulators in intestinal epithelial cells (IECs) in turn. An anti-inflammatory mechanism of commensal *B. thetaiotaomicron* has been founded. *B. thetaiotaomicron* weakens proinflammatory cytokine expression level in IECs via contributing to nuclear translocation of the NF-κB subunit RelA, by a PPARγ-dependent pathway [34]. Another example is that *L. jensenii* TL2937 down-regulates LPS-triggering proinflammatory cytokines and chemokines production in PIE cells via enhancing the expression of A20, Bcl-3 and MKP-1, which retard the TLR4-dependent NF-κB and MAPK activation [9]. Moreover, continuously up-regulation of A20, Tollip, and SIGIRR plays a major part in the anti-inflammatory activity of *L. casei* MEP221114 in poly(I:C)-stimulated PIE cells [35]. The increased level of TLRs pathway negative regulators can also be found in other LAB which can alleviate inflammatory damages, such as *L. plantarum* [21], Ld, APS and NPS [28]. Consistently, the results here showed that *L. acidophilus* obviously up-regulated mRNA expression level of negative regulator Tollip and Bcl-3 in spleen post ETEC infection. Thus, Tollip and Bcl-3 are of importance in the anti-inflammatory effect of *L. acidophilus*. Interestingly, we found that A20, Tollip, Bcl-3 and IRAK-M were all up-regulated without the challenge by ETEC. The findings indicated that the uptake of *L. acidophilus* from diets resulted in a higher level of negative regulators of TLRs signaling pathways, thus to establish an anti-inflammatory state against inflammation damage.

## Conclusions

In summary, *L. acidophilus* suppress the activation of the different steps of NF-κB and MAPK signaling in piglets, by inhibiting ETEC-induced TLR2 and TLR4 expression, impaired the phosphorylation of NF-κB and MAPK leading to reduce the production of proinflammatory cytokines, as well as up-regulating negative regulators and anti-inflammatory cytokines. Taken together with the results presented here, we speculate that *L. acidophilus* alleviates inflammatory response to ETEC via inhibition of the NF-κB and mitogen-activated protein kinase signaling pathways in piglets. This underlying mechanism provides a theoretical basis for the clinical application of lactic acid bacteria.

## Abbreviations

ETEC: Enterotoxigenic *Escherichia coli*
IL: Interleukin
*L. acidophilus*: *Lactobacillus acidophilus*
MAPK: Mitogen-activated protein kinase
MLNs: Mesenteric lymph nodes
NF-κB: Nuclear factor kappa B
TLR: Toll-like receptor
